# Weighted simulations redefining quadratic calibration: democratisation of analytical uncertainty evaluation

**DOI:** 10.1007/s00216-026-06638-4

**Published:** 2026-06-30

**Authors:** Felipe Rebello Lourenço, Carla Palma, Carlos Borges, Elcio Cruz de Oliveira, Ricardo J. N. Bettencourt da Silva

**Affiliations:** 1 Faculdade de Ciências Farmacêuticas, Universidade de São Paulo, Av. Prof. Lineu Prestes, 580 – Bloco 15, São Paulo, SP 05508-000 Brazil; 2 Instituto Hidrográfico, Rua das Trinas, 49, 1249-093 Lisbon, Portugal; 3 Postgraduate Programme in Metrology, Pontifical Catholic University of Rio de Janeiro, Rio de Janeiro, 22451-900 Brazil; 4 Land Transportation and Storage, Measurement and Product Inventory Management, Logistics, Petrobras S.A., Rio de Janeiro, 20231-030 Brazil; 5 Centro de Química Estrutural, Institute of Molecular Sciences, Departamento de Química e Bioquímica, Faculdade de Ciências, Universidade de Lisboa, Campo Grande, 1749-016 Lisbon, Portugal

**Keywords:** Quadratic regression, Weighted regression, Uncertainty evaluation, Monte Carlo simulations, Weighted simulations

## Abstract

Instrumental methods of analysis are often calibrated across broad analyte level ranges. Nevertheless, instrumental responses over such extended ranges are frequently nonlinear and heteroscedastic. Accurately assessing these responses, particularly the uncertainty associated with quantifications derived from calibration curves under these conditions, is challenging. Although simpler models may appear attractive, they require more assumptions that can be difficult to verify, even when suitable software is available, which considerably limits their widespread application. This study presents a weighted simulation method that enables reliable and straightforward weighted regression of quadratic relationships between instrumental responses and calibrator concentrations, while also supporting the evaluation of uncertainty from quantifications obtained in such regressions. The proposed tool requires only that the instrumental response follows a quadratic function and that uncertainty in calibrator values is negligible. The algorithm’s simplicity is achieved by replicating simulation lines in proportion to the inverse of the signal variance. The method was successfully tested using artificially generated instrumental responses exhibiting a quadratic dependence on analyte concentration and heteroscedastic variance. The approach produced accurate regression coefficients and analyte concentration estimates for unknown samples with low associated uncertainty. Furthermore, the method is potentially applicable to other types of regression and has been implemented in a user-friendly MS Excel spreadsheet. This work aims to democratise access to accurate weighted quadratic regression and uncertainty evaluation for quantifications based on such calibrations, thereby contributing to the improvement of measurement quality in chemistry performed in conformity assessment and research and development.

**Graphical Abstract:**

**Figure:**
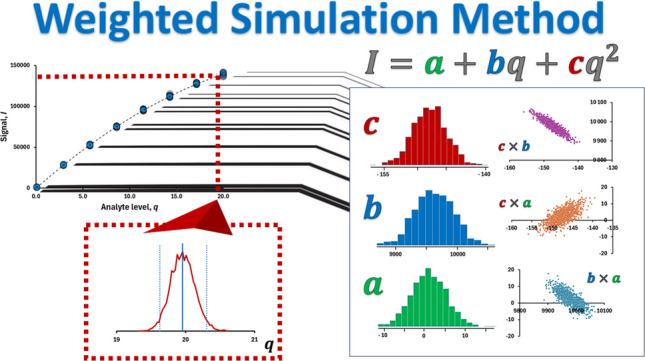

**Supplementary Information:**

The online version contains supplementary material available at 10.1007/s00216-026-06638-4.

## Introduction

New and more advanced instrumental methods of analysis are stretching the lower and upper limits of analyte levels that can be identified and quantified in a reliable way. However, wider analytical intervals are frequently associated with nonlinear instrumental responses often modelled by quadratic regressions. Typically, together with the nonlinearity, signals are heteroscedastic (i.e. are associated with significantly different precision), making the accurate modelling of instrumental response and of quantification from these calibrations challenging. Analysts can always divide the analytical interval into shorter, approximately linear intervals, trading off the management of a single complex model for a series of contiguous linear models with increasing inconsistencies as the linear segments grow wider.

In the 1970 s, Schwartz conducted several studies related to calibration curves. In one of them, Schwartz presented methods to handle calibration curves when measurement variance varies along the curve, addressing nonuniform precision in analytical data. Two examples—photometric and chromatographic analyses—illustrate how to model and calculate confidence limits under heteroscedastic conditions. The findings highlight the importance of accounting for variance changes to improve statistical reliability in quantitative assays [1]. In another paper, Schwartz discussed methods for calibrating nonlinear instrument response curves, emphasising polynomial fitting and uncertainty evaluation. It highlights challenges in accurately determining unknown values due to curve curvature and proposes Monte Carlo simulations to assess error propagation. The approach improved confidence in measurements where linear calibration was insufficient [2]. In 1977, a paper addressed the challenge of determining confidence intervals for analyses derived from nonlinear calibration curves. It compares three computational methods—linear segments, three-parameter functions, and polynomial fitting—to evaluate uncertainty [2].

Three decades ago, a study presented a comprehensive strategy for validating calibration models in atomic absorption spectrometry, emphasising experimental design and statistical tests. It highlights the importance of detecting outliers, assessing variance behaviour, and evaluating model fit to ensure accurate analytical results. Recommendations were provided for both linear and nonlinear calibration approaches to enhance method reliability. The simulation of data based on real measurements was used to provide an accurate assessment of evaluation procedures [3].

Morgado et al. developed a tool for the Monte Carlo Method simulation of calibrations and sample signal interpolation in the calibration curve applicable to heteroscedastic signals and cases where calibrators’ values are affected by relevant uncertainty. This approach involves modelling precision variation with the concentration and was applied to simulate calibrator values and signals, enhancing the accuracy of the calibration and uncertainty estimation for elements such as As, Cd, Ni and Pb [4].

Oliveira and Aguiar presented a validated methodology for uncertainty evaluation in calibration curves fitted with second-degree polynomials, using both the GUM approach and Monte Carlo simulations. The quadratic model provides a superior fit and more accurate uncertainty estimates compared to linear models. Results confirm the method’s reliability for analytical measurements with homoscedastic data and negligible calibration standard uncertainties [5].

Kirkup and Mulholland compared linear, quadratic, and nonlinear calibration models for HPLC data, highlighting improved accuracy with nonlinear approaches. Statistical analyses, including Akaike’s information criterion (AIC) and adjusted R^2^, demonstrated better fit and reduced prediction errors for nonlinear equations. The findings suggested considering nonlinear calibration when slight curvature appears in analytical data [6].

The ISO 8466–1:2021 standard outlines various calibration methods for chemical and physicochemical analyses, emphasising linear calibration functions. It covers procedures for establishing calibration ranges, calculating calibration functions, and validating calibration accuracy. The document also addresses strategies to manage matrix effects and measurement uncertainties in water quality testing [7]. On the other hand, ISO 8466–2:2001 outlines a method for calibrating and evaluating analytical procedures using second-order polynomial functions when linear calibration is insufficient. It provides guidelines for establishing working ranges, testing variance homogeneity, calculating calibration coefficients and determining confidence intervals for analytical results. This standard is primarily intended for method development in water quality analysis [8].

Malengo and Pennecchi presented a versatile MATLAB-based weighted total least squares (WTLS) algorithm capable of handling calibration problems with uncertainty for correlated variables and any fitting model. The software developed, designated CCC for “Calibration curve computing”, offers improved parameter estimation accuracy and uncertainty evaluation, validated through benchmark datasets and Monte Carlo simulations. The method supports complex covariance structures, enhancing calibration curve fitting beyond traditional approaches. The covariance matrices can subsequently be used to estimate the values of the analysed unknown items, including the measurement uncertainty, requiring additional software [9, 10].

In 2021, Zhang addressed nonlinear regression with multiplicative measurement errors affecting both response and predictors. It introduces a second-order least squares estimator incorporating conditional moments to improve parameter estimation. Simulation results demonstrated that applying calibration methods enhances estimator accuracy compared to traditional approaches [11].

Klauenberg et al. developed informative Bayesian priors to enhance ELISA test analysis, improving concentration estimates under nonlinear heteroscedastic regression. The proposed priors were validated through simulations and real data from various labs and analytes, showing robustness and reduced uncertainty. These priors offered a practical tool for more reliable immunoassay inference and encouraged broader application to similar assays [12].

Sadraya et al. demonstrated that, when both heteroscedasticity and nonlinearity were present in methotrexate plasma concentration measurements, generalised least squares regression with variance function estimation outperforms traditional weighted and ordinary least squares methods. The approach yielded more accurate calibration models and drug concentration estimates over a wide dynamic range. Consequently, it enhanced reliability in pharmacokinetic analyses involving methotrexate [13].

Liu et al. discussed linear fitting results, comparing slope and intercept estimates obtained by different robust weighted total least squares methods. They highlighted that the proposed median parameter-based approach achieved more stable and accurate linear fitting, especially in the presence of multiple gross errors, demonstrating improved resistance to outliers compared to traditional methods [14].

Zhou and Fang introduced a mixed weighted least squares and weighted total least squares method designed for error-in-variables models with fixed and random design matrix columns. The proposed approach enhanced computational efficiency without sacrificing accuracy, particularly for large or sparse matrices. Applications in geodesy, such as 3D coordinate transformations and parallel line fitting, demonstrated its effectiveness [15].

Finally, Balsamo et al. analysed uncertainty propagation in nonlinear parameter estimation, revealing that traditional Hessian-based methods often underestimate uncertainty. They proposed a unique, minimisation-independent approach based on the estimator’s Jacobian, providing more accurate uncertainty estimates, especially demonstrated in total least-squares line fitting [16].

The research conducted so far on evaluating the uncertainty from quadratic regressions involved the development of complex tools supported by a list of assumptions inversely proportional to model complexity. The development of adequate software simplified the use of such tools. However, model complexity is always a limitation for their wide use because analysts do not feel comfortable using tools they do not fully understand.

This research developed an accurate and easy-to-follow methodology for evaluating the uncertainty from quantifications based on inverse interpolation of weighted quadratic regressions. The weighted Monte Carlo simulation of signals, which runs signals' simulations in a number inversely proportional to their precision, allows estimating model uncertainty equivalently to complex algorithms that require additional assumptions. The easy understanding of how algorithms perform enables their generalised use in chemical analysis performed to take a decision with relevant socio-economic impact, such as the conformity or price of products. The approach was validated using simulated signals that vary quadratically with the level of the quantity under study and have known true regression parameters, to accurately assess their performance. The principles of the Weighted Simulation Method suggest it can be applied to other types of nonlinear functions. The limitation of requiring computational resources for simulation are becoming less significant as more efficient software and computers become widely available, inexpensive and fast.

## Materials and methods

### Weighted simulation method

The designated "Weighted simulation method" involves determining the imprecision of the instrumental signal or indication [17], ${I}_{i}$, at least, at six [3], N, (i=1 to N) equidistant analyte levels, ${q}_{i}$, (q stands for quantity that can be a concentration or mass fraction) within the instrumental calibration interval. The signal’s imprecision is quantified by the signal’s standard deviation, ${s}_{I(i)}$, from ${n}_{i}$ replicates, being the weighing factor, ${w}_{i}$, associated with each ${q}_{i}$ determined by Eq. (1). Replicate signals from the same calibrator are expected since the calibrator’s values have a negligible uncertainty (i.e. independently prepared calibrators are equivalent).

1 $${w}_{i}=\frac{\left[{s}_{I(i)}^{-2}\cdot {n}_{i}\right]\sum_{i=1}^{N}{n}_{i}}{\sum_{i=1}^{N}\left[{s}_{I(i)}^{-2}\cdot {n}_{i}\right]}$$

**Fig. 1:**
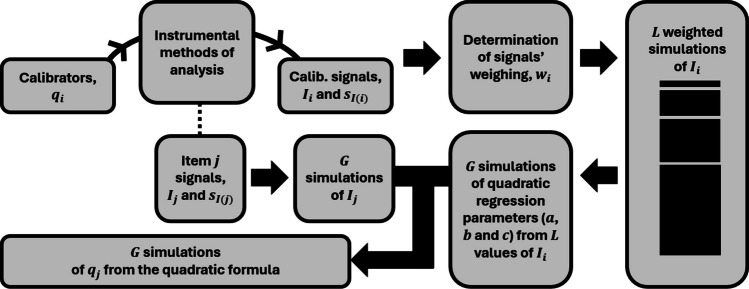
Schematic description of weighted simulation of the analyte value, ${q}_{j}$, of item *j* from the inverse interpolation of their signal in a calibration curve built by the weighted quadratic regression

For a total of L Monte Carlo Method simulations, $\left[L{w}_{i}/\sum_{i=1}^{N}{w}_{i}\right]$ replicate simulations of signals from each level, ${q}_{i}$, considering the mean signal, ${\overline{I} }_{i}$, and their standard deviation, ${s}_{I(i)}$, are performed. For each weighted simulation of L lines, the quadratic model parameters (i.e. a, b and c from the relation $I=a+bq+c{q}^{2}$) are determined from the unweighted quadratic regression model [8]. The weighing is achieved by simulating signals in a number inversely proportional to their variance. The determination of these parameters is then replicated G times, from which a correlation matrix, R, and standard deviations of a, b and c are determined to allow assessing the weighing. From the replicate signals of the item j to be quantified using the calibration curve, it is determined the mean signal, ${\overline{I} }_{j}$, and their standard deviation, ${s}_{I(j)}$, afterwards used to simulate G signals of the item j. For each simulated ${I}_{j}$, a, b and c for G times, analyte values in the item j are determined by using the well-known quadratic formula, producing the same number of ${q}_{j}$ estimates (i.e. analyte level in the unknown item *j*). Percentiles of simulated ${q}_{j}$ values are used to determine confidence limits for the estimate, considering the selected confidence level. Figure 1 presents a schematic description of the developed Weighted Simulation Method. This method was assessed by comparing it with well-established alternatives implemented in validated software, namely the approach implemented in software by Lecuna et al. [10] (i.e. the latest version of “CCC” software). This comparison was performed using artificially simulated instrumental signals that allow an accurate comparison of estimated regression parameters with the known true values and an objective comparison of various evaluation approaches. The expected deviation of real instrumental signals from a quadratic variation with the studied quantity, or the occasional occurrence of outlier signals, would make the uncertainty evaluated by the various tools equivalent due to unbalanced or outlying residuals. Therefore, this work does not discuss how real data inconsistencies affect measurement uncertainty evaluations, although increased uncertainty values are expected.

## Results and discussion

### Simulated scenarios

Table 1 presents the studied artificial scenarios regarding the type of variation between the instrumental signal and the analyte level (linear or quadratic) and between the signal’s precision and the analyte level (homoscedastic or heteroscedastic, i.e. constant or varying precision in the calibration interval). Increasing signals and, for heteroscedastic cases, increasing signal’s standard deviation with the analyte level are considered. Scenarios A and B, and C to F represent linear and quadratic relations, respectively. On the other hand, scenarios A and C, and B, D, E and F represent homoscedastic and heteroscedastic signals, respectively. Quadratic regression models work well in linear relations, producing a quadratic term equivalent to zero. The studied scenarios are intended to assess how evaluation performs under increasingly complex signal variations (i.e. from linear to quadratic, and from homoscedastic to heteroscedastic) and highly precise signals, to allow better differentiation of the outputs of the compared approaches. An extreme, unrealistic, non-univocal quadratic relationship was examined to stretch the comparison of approaches (Scenario F).

**Table 1:** Studied artificial instrumental signal scenarios where I and q represent the signal and analyte level, respectively

	Modelling		Calibration levels	Replicate signals
Case	Ivs.q	${s}_{I}$vs.q	❶|❷|❸|❹|❺|❻|❼|❽	
A	I=0+10000q	${s}_{I}=1$	0.100|2.943|5.786|8.629| 11.471|14.314|17.157| 20.000	10 at each level
B	I=0+10000q	${s}_{I}=0.01q$		
C	$I=0+10000q-150{q}^{2}$	${s}_{I}=1$		
D	$I=0+10000q-150{q}^{2}$	${s}_{I}=0.01q$		
E	$I=0+10000q-150{q}^{2}$	${s}_{I}=0.01q$		5 at each level
F	$I=0+10000q-498{q}^{2}$	${s}_{I}=0.01q$		10 at each level

A–Linear and homoscedastic; B–Linear and heteroscedastic; C–Quadratic and homoscedastic; D, E and F–Quadratic and heteroscedastic

The CCC software developed by Lecuna et al. [10] takes calibrators’ signals and their covariance matrix to estimate the parameters from the weighted quadratic regression (a, b and c) and their standard deviations and covariance matrix, thus requiring the subsequent use of this matrix and a model of the signal from the analysed unknown item for the determination of the analyte level in the unknown item. Therefore, the comparison between the performance of the developed Weighted Simulation Method (WSM) and the CCC [10] is only direct when the regression parameters and their standard deviations and the correlation matrix are compared. In order to extend the comparison between WSM and CCC to the estimate of the analyte level of an unknown item, two extensions of the CCC model were considered: one using the Monte Carlo Method (CCC-MCM) and a second using the Kragten method [18] (CCC-Kragten) for combining uncertainty components. For the CCC-MCM, a simulator of correlated regression parameters (a, b and c) from the covariance matrix produced by CCC was used. The simulator was based on a previously developed algorithm used to study correlated variables involving the Cholesky decomposition of the covariance matrix [19].

The CCC-Kragten takes the correlation matrix between ${I}_{j}$, a, b and c considering negative or positive increments of variables’ uncertainty with a magnitude equivalent to their standard deviation. The ${I}_{j}$ is independent of a, b and c, which are correlated with each other. The Kragten method fails to handle eventual linearity deviations between the estimated analyte level of an unknown item and its input quantities (i.e. ${I}_{j}$, a, b and c). In fact, only the WSM completely handles linearity deviation from the estimate of the measured value and its input quantities. For instance, if the quadratic term, c, has an asymmetric distribution, it will impact on estimated analyte level of an unknown item; an impact which cannot the considered by the CCC-MCM or CCC-Kragten method, which assumes c has a normal distribution. Equivalent estimates from negative and positive increments in CCC-Kragten were observed in the studied examples (A to D), indicating the linearity of the estimated analyte level in the unknown item, considering their input quantities [20].

**Fig. 2:**
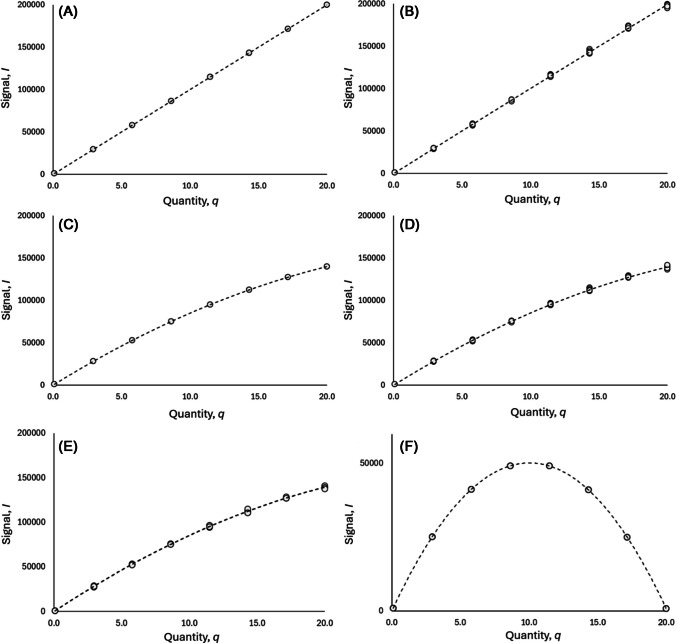
Calibration curves for (**A**) linear homoscedastic model, (**B**) linear heteroscedastic model, (**C**) quadratic homoscedastic model, (**D**), (**E**) and (**F**) quadratic heteroscedastic model

Figure 2 presents the graphical representation of the simulated signal and the regression line from the six studied scenarios. Highly precise signals were simulated to avoid masking linearity or curvature from a lack of precision.

Table 2 presents the regression parameters, their standard deviation and correlation matrices estimated by the WSM and CCC. The covariance matrix is built from the previous information. Both tools estimate regression parameters equivalent to the known value presented in Table 1 for a 95% confidence level, proving the accuracy of the estimates. The parameters from WSM and CCC are also equivalent to each other for the same confidence level. Regarding standard deviations of regression parameters, considering the first four Scenarios (i.e. A to D), from the 12 compared pairs WSM vs CCC, only two are statistically different for a 99% confidence level (regression parameters a and b from Case B). In the studied cases, the CCC is always more precise than the WSM. The correlation matrices estimated by both tools are equivalent, proving that they handle signals weighing equivalently. In order to additionally assess the performance of both regression models, two extra case studies were evaluated: Case E: Quadratic variation of instrumental signal described with fewer (i.e. 5) replicate signals and Case F: Extreme and unrealistically multivalued (i.e. not biunivocal) instrumental response. For these cases, regression parameters are statistically equivalent to the known values and each other, but CCC is significantly more precise in both scenarios.

**Table 2:**
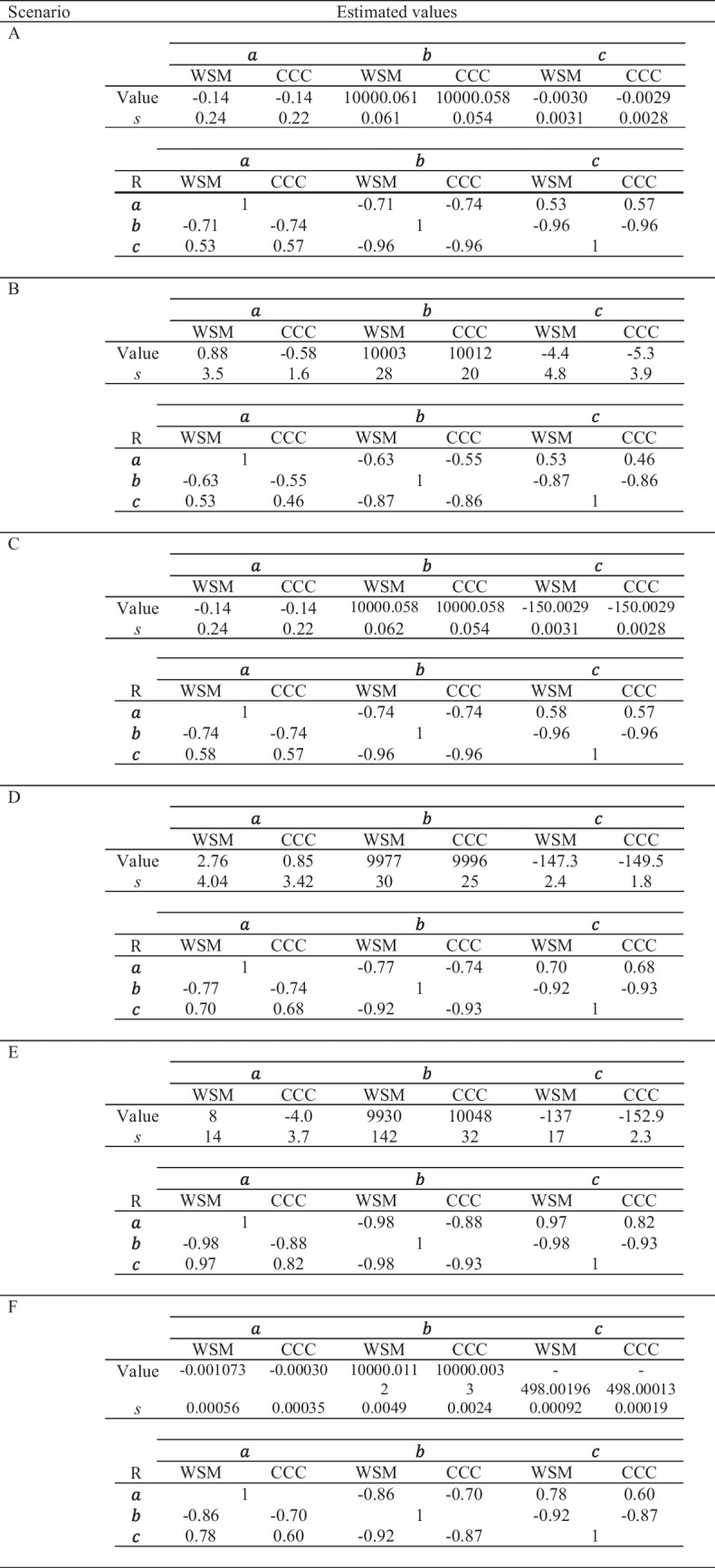
Comparison of regression lines estimated by the Weighted Simulation Method (WSM) and CCC software for the studied instrumental signal scenarios specified in Table 1. The value and standard deviation, *s*, of the regression parameters (a, b and c; $I=a+bq+c{q}^{2}$) and their correlation matrix, $\mathrm{R}$, are presented

The CCC estimates equivalent standard deviations of regression parameters for cases D and E associated with 10 and 5 replicate signals of calibrators, while these standard deviations increase significantly for five replicates in WSM determinations. This observation results from the fact that CCC does not take the degrees of freedom of the signal’s covariance matrix in the regression, which is a relevant limitation when a reduced number of replicate signals are available. The relevance of this limitation, which depends on the specific scenario, was not studied in this work.

Table 3 presents the estimates of analyte values performed by the three models (WSM, CCC-MCM and CCC-Kragten) for the first four Scenarios (i.e. A to D) from Table 1 where the analysed items S1 to S5 have levels and signals equivalent to the calibrator’s ones, and ten replicate signals were considered. All estimates are compatible with reference values (0.100000; 8.628571; 11.471429; 17.157143 or 20.000000) known without any uncertainty for 95% confidence levels, except for two estimates by CCC-Kragten method. The WSM, CCC-MCM and CCC-Kragten produce confidence intervals associated with relatively equivalent relative half ranges that vary between 0.00041%—1.7%, 0.00048%—1.7% and 0.00037%—1.8%, respectively. The precision gains from regression parameters observed in CCC are not reflected in the analysis of unknown items. The absence of uncertainty in reference values and the high precision of the signals would enable better identification of model inconsistencies.

**Table 3:** Confidence intervals, for 95% confidence level, estimated for Scenarios A to D, by the WSM, CCC-MCM and Kragten methods for the analyte level in "unknown" items S1 to S5 with value equivalent to calibrators, i.e. 0.100000; 8.628571; 11.471429; 17.157143 and 20.000000. Confidence intervals are reported with two uncertain figures. On the right side of the interval, it is reported the equivalence (EQ) and difference (DF) (marked in bold) with the known value that has no uncertainty

		WSM	CCC-MCM	Kragten
A	S1	[0.099943; 0.100075] EQ	[0.099962; 0.100069] EQ	[0.099890; 0.100039] EQ
	S2	[8.628447; 8.628642] EQ	[8.628342; 8.628662] EQ	[8.628496; 8.628660] EQ
	S3	[11.471350; 11.471525] EQ	[11.471346; 11.471522] EQ	[11.471266; 11.471542] EQ
	S4	[17.157033; 17.157173] EQ	[17.156983; 17.157156] EQ	**[17.157013; 17.157140] DF**
	S5	[19.999944; 20.000140] EQ	[19.999989; 20.000182] EQ	[19.999955; 20.000122] EQ
B	S1	[0.09951; 0.10125] EQ	[0.09950; 0.10140] EQ	[0.09955; 0.10107] EQ
	S2	[8.528; 8.694] EQ	[8.475; 8.699] EQ	[8.498; 8.700] EQ
	S3	[11.382; 11.591] EQ	[11.425; 11.564] EQ	[11.356; 11.531] EQ
	S4	[16.899; 17.223] EQ	[17.022; 17.210] EQ	[16.932; 17.265] EQ
	S5	[19.78; 20.26] EQ	[19.93; 20.26] EQ	[19.90; 20.31] EQ
C	S1	[0.099943; 0.100076] EQ	[0.099941; 0.100090] EQ	[0.099950; 0.100066] EQ
	S2	[8.62840; 8.62867] EQ	[8.62845; 8.62876] EQ	[8.62840; 8.62871] EQ
	S3	[11.471307; 11.471573] EQ	[11.471281; 11.471550] EQ	[11.471393; 11.471561] EQ
	S4	[17.15691; 17.15720] EQ	[17.15687; 17.15734] EQ	[17.15694; 17.15721] EQ
	S5	[19.99987; 20.00035] EQ	[19.99991; 20.00036] EQ	[20.00000; 20.00047] EQ
D	S1	[0.09921; 0.10061] EQ	[0.09894; 0.10015] EQ	[0.09921; 0.10072] EQ
	S2	[8.520; 8.714] EQ	[8.502; 8.687] EQ	[8.531; 8.682] EQ
	S3	[11.37; 11.61] EQ	[11.24; 11.63] EQ	[11.30; 11.58] EQ
	S4	[16.78; 17.17] EQ	[16.88; 17.22] EQ	**[16.66; 17.10] DF**
	S5	[19.62; 20.31] EQ	[19.86; 20.43] EQ	[19.45; 20.15] EQ

The data collected proves the equivalence of estimates performed by the WSM and CCC methods. However, the WSM method has the advantage of considering the degrees of freedom of collected calibrators’ and analysed samples’ signals, can handle the relevant lack of linearity of the measurement function and is easy to understand and follow by any analyst. The characteristics of the algorithm’s performance that should be prioritised depend on the specific scenario under study. For instance, if only a small number of replicate signals is available, robustness to limited data is important, particularly for imprecise signals. The ability to handle asymmetry in the estimated distribution of regression parameters is also important when this asymmetry is relevant to the measured values.

The only assumption WSM and CCC methods require is the adjustment of the instrumental signal to a quadratic response and the negligible uncertainty of calibrators’ values that can be easily checked using adequate statistical tools [21]. The fitness of the quadratic model to signal’s variation with the studied quantity can be assessed by the χ^2^-test described by the Analytical Methods Committee [22] implemented in a spreadsheet by Pluháček et al. [21, 23]. The assessment of the negligible uncertainty of calibrators’ values involves proving that the standard uncertainty of the ratio of the value of any pair of calibrators is not larger than one third or one fifth of the standard deviation of the ratio of respective signals. This test is also described and was implemented in a spreadsheet by Pluháček et al. that covers all uncertainty components from calibrator’s preparation [21, 23]. The same authors have discussed how the signal’s weighting can be defined, even if no replicate signals of the analysed item are available [21].

The WSM was implemented in a user-friendly MS Excel file, made available as Supplementary Material, having the only disadvantage of requiring around 5 min for 10000 simulation lines on an updated PC. The adequacy of the number of performed simulations is tested by checking the agreement of replicate simulations. For most analytical applications, 20% differences between simulated parameters are adequate. Simulations can be much faster if implemented on optimised platforms, such as those based on Python algorithms.

## Conclusions

The developed transparent and easy-to-follow algorithm for weighted quadratic regressions applicable to instrumental responses that fit these functions was successfully applied to several artificially simulated scenarios of highly precise signals. Transparency comes from algorithmic simplicity that can be followed and understood by someone with a basic background in mathematics.

Cases where the signal varies linearly and quadratically with the analyte levels, with homoscedastic or heteroscedastic signal’s variance, were tested, being estimated regression parameters equivalent to the known values that have no uncertainty. When these models were used to estimate known analyte levels from simulated signals, they produced equally accurate estimates. The performance of the developed Weighted Simulation Method was compared with the validated Software CCC based on Weighted Least Squares. Regression parameters estimated by CCC were equivalent to those estimated by WSM but more precise because it does not take degrees of freedom of the estimated signal’s precision into account. Since the CCC software only estimates regression parameters and their covariance matrix, Monte Carlo Method and Kragten methods extension of this tool was developed for the determination of analyte level in unknown items with uncertainty. Although not relevant in the studied scenarios, the WSM is the only of the assessed approaches that accurately handles any lack of linearity between regression model estimates and their input quantities. The principles of the developed approach suggest it could be applicable to other nonlinear relationships between the instrumental signal and the quantity under study, and to cases where calibrator values have significant uncertainty, although this was not assessed in this work. The only drawback of the developed approach is the computational resources required, a problem which can be overcome by using updated computers with optimised software such as Python. The MS Excel tools used in this work are made available as Supplementary Material. The authors believe the developed tool can contribute to widespread accurate management of complex regression models.

## Supplementary Information

Below is the link to the electronic supplementary material.

Supplementary file1 (XLSM 21.2 MB)

## Data Availability

All data generated or analysed during this study are included in this published article and its accompanying supplementary information files.
